# Prenatal Diagnosis of Hemimegalencephaly Using Radiological Methods: A Case Report

**DOI:** 10.7759/cureus.54456

**Published:** 2024-02-19

**Authors:** Marcia Mejia, Santiago Vargas Arango, Sergio Vargas Vélez, Jonathan Pimiento Figueroa

**Affiliations:** 1 Radiology, Universidad de Antioquia, Medellin, COL; 2 General Medicine, Universidad CES, Medellin, COL; 3 Neuroradiology, CediMed, Medellin, COL; 4 Radiology, Hospital Universitario de San Vicente Fundación, Medellin, COL

**Keywords:** malformations of cortical development, congenital abnormalities, magnetic resonance imaging, prenatal diagnosis, hemimegalencephaly

## Abstract

Hemimegalencephaly is a rare congenital anomaly characterized by an increase in the size and dysplastic involvement of one cerebral hemisphere, which can be partial or complete. It may also be associated with anomalies in the cerebellum and brainstem and, in some cases, be a part of different syndromes. The result of these abnormalities leads to intractable epilepsy and developmental delay. Diagnosis is typically made through imaging studies in the postnatal period, but it can also be done before birth. We present the case of a 23-week pregnant patient in whom a prenatal diagnosis of hemimegalencephaly was made, highlighting the need for fetal magnetic resonance imaging (MRI) to confirm the diagnosis.

## Introduction

Hemimegalencephaly is a rare congenital anomaly of cortical development that can occur in isolation or be associated with different syndromes. It has an equal prevalence in both sexes, with one to three cases per 1,000 epileptic children [1]. It is characterized by an enlargement and dysplasia of one of the cerebral hemispheres with partial or total involvement. The affected hemisphere shows a variable degree of alterations in gyration, cortical malformations, and dilation of the ventricular system, which may or may not be accompanied by alterations in the cerebellum and brainstem.

Patients present with intractable epilepsy and psychomotor developmental delay. The usual treatment is hemispherectomy for the control of seizure events [1-4]. This condition was first mentioned in the literature in the 1830s [2] based on autopsy reports. However, in more recent decades, its findings have been described in imaging with several case reports of prenatal diagnosis through ultrasound and fetal magnetic resonance imaging (MRI) [3-7].

This case shows the typical imaging findings during the prenatal period and also highlights some MRI findings that have not been described so frequently in this condition. Consequently, the confusion in the diagnosis that can be generated due to the low frequency of this pathology can be reduced.

## Case presentation

**Figure 1:**
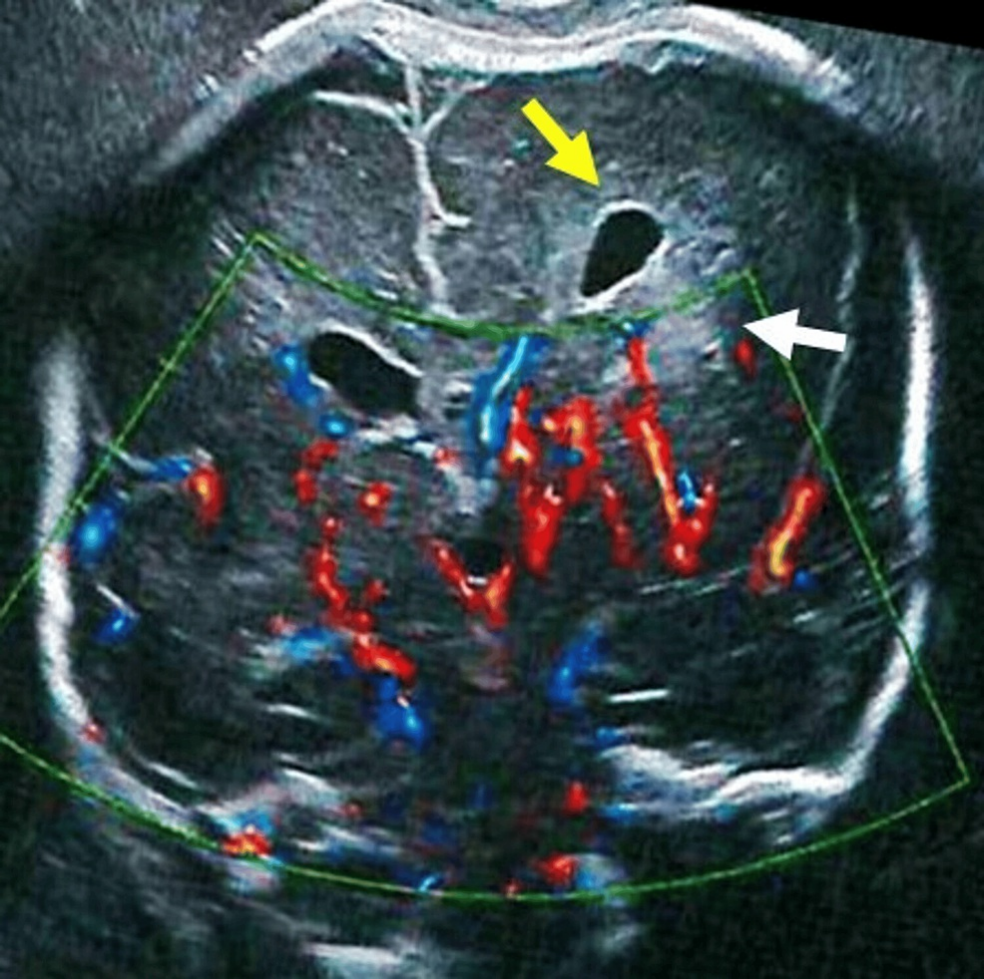
Prenatal ultrasound, coronal section of the brain. Dilatation of the left-sided lateral ventricles (yellow arrow), with hyper-echogenicity of the adjacent white matter (white arrow).

A 33-year-old pregnant woman underwent a routine obstetric ultrasound at 23 weeks of gestation, which revealed left unilateral ventriculomegaly with a mild midline shift. A follow-up ultrasound at 26 weeks showed persistent unilateral ventriculomegaly, associated with midline shift and increased echogenicity of the brain parenchyma on the same side (Figure 1). These findings were interpreted as an intraparenchymal hemorrhage with mass effect.

**Figure 2:**
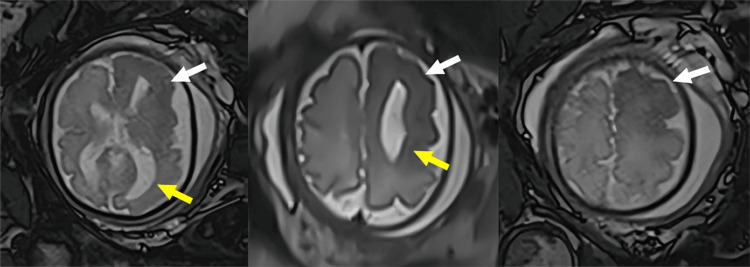
Fetal magnetic resonance imaging, T2 sequence, axial sections. Increased size of the left cerebral hemisphere, with dilatation of the left lateral ventricle (yellow arrow), alteration in corticosubcortical differentiation and hypointensity of the white matter (white arrow).

The patient was advised to undergo fetal MRI, which was performed at 30 weeks of gestation, revealing enlargement of the left cerebral hemisphere with predominant ventriculomegaly on the same side and midline shift to the right (Figure 2).

**Figure 3:**
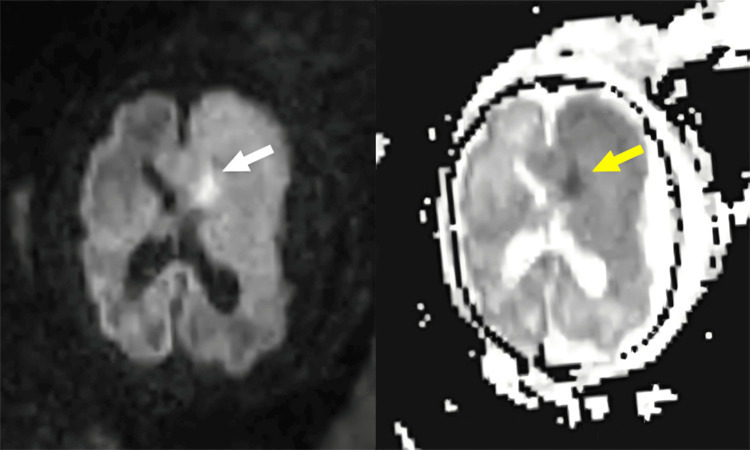
Fetal magnetic resonance imaging, diffusion sequences (DWI, ADC). Tissue diffusion restriction in the left frontal region, indicated by hyperintensity in DWI (white arrow) and hypointensity in ADC (yellow arrow). DWI: diffusion-weighted imaging, ADC: apparent diffusion coefficient

In addition, there was a differentiation alteration between the left frontal gray and white matter, with hypointensity in the T2 sequence and focal tissue diffusion restriction (Figure 3). This was accompanied by frontal cortical thickening with areas of pachygyria and polymicrogyria (Figure 2).

Taken together, these findings were interpreted as left hemimegalencephaly. The mother received counseling from a multidisciplinary team and chose to terminate the pregnancy in accordance with the current legislation in Colombia.

## Discussion

In the normal development of the brain, there are two major processes: first, the closure of the neural tube occurs, followed by the formation of the cerebral hemispheres and the corpus callosum; second, the formation of the cortex takes place. The latter involves the processes of proliferation, migration, and organization, which mainly occur simultaneously between the 12th and 20th weeks of gestation [8].

Hemimegalencephaly is a rare condition, with an equal prevalence in both sexes and one to three cases per 1,000 epileptic children. It is characterized by an increase in the size of one cerebral hemisphere, associated with partial or total dysplasia of the parenchyma. It presents with variable degrees of pachygyria, polymicrogyria, or lissencephaly. Rarely, it is associated with an increase in the size of the cerebellum, brainstem, and corpus callosum on the affected side [9].

This condition can occur in isolation or be associated with different syndromes (especially neurocutaneous) and is classified into different types. Type 1 is characterized by isolated involvement, which clinically manifests as seizures and whose prognosis depends on the degree of associated cortical alterations. Type 2 is associated with different syndromes (epidermal nevus syndrome, Proteus syndrome, neurofibromatosis, tuberous sclerosis, and Klippel-Trenaunay-Weber syndrome) and may be accompanied by homolateral hemihypertrophy. Type 3 presents an increase in the size of the cerebellum and brainstem on the same side as the affected cerebral hemisphere [2].

In the pathophysiology, different mechanisms have been described, including alterations in the processes of neuronal proliferation, differentiation, or migration. This is associated with mutations in the genes involved in the mammalian target of rapamycin (mTOR) signaling pathway, with the PIK3CA gene being the most frequently affected [1,2,8]. Histologically, it manifests as disorganized cytoarchitecture of the cortex and subcortical white matter, along with heterotopias and alterations in lamination [3].

Clinically, patients present with intractable epilepsy that begins in the first six months of life, developmental delay, psychomotor deficits, and progressive hemiparesis. Treatment consists of hemispherectomy for total or partial control of seizure events; however, it is common for there to also be alterations in the contralateral hemisphere that cause the persistence of seizures. Anatomic hemispherectomy has shown better results in controlling seizure events, but it presents a higher risk of complications, so in many cases, the functional technique is preferred [4,10].

This pathology can be detected prenatally; however, some case reports mention that imaging findings evolve during the prenatal period, making interpretation more difficult in the early stages of gestation. Ultrasound is usually the initial examination, but fetal magnetic resonance imaging is required to confirm the diagnosis and properly characterize the abnormalities [1,4].

Ultrasound is the preferred diagnostic method during pregnancy, but certain technical conditions can reduce its effectiveness and lead to some abnormalities being missed. Fetal MRI is used in specific cases where ultrasound is insufficient or abnormalities are not fully visible, usually carried out after the 22nd week of gestation. Some factors that can reduce the effectiveness of ultrasound include anterior placenta, oligohydramnios, interference from intestinal gas or other anatomical structures, and large maternal body size, among others. There are also several specific indications for fetal MRI, particularly abnormalities in the central nervous system; some of these include ventriculomegaly, absence of the cavum septum pellucidum, malformations of the posterior fossa, and neural tube defects, among others [11].

The main findings in ultrasound are an increase in head circumference (above the 90th percentile), unilateral ventriculomegaly, asymmetry in the cerebral hemispheres with displacement of the midline, cortical thickening, and alteration in the pattern of sulci [1,4,5].

In MRI, the findings include an increase in the size of one cerebral hemisphere, asymmetric dilation of the ventricular system, thickening and dysplasia of the cortex (polymicrogyria, lissencephaly, agyria, pachygyria, and heterotopias), dystrophic calcifications of the parenchyma, and alteration in the differentiation between gray and white matter. An alteration in the signal of the white matter has been described, presenting changes over time: in the first year of life, it is hyperintense in T1 and hypointense in T2, and later it presents an appearance of alternating hypo- and hyperintense bands. Tissue diffusion restriction in affected areas has also been described due to its high cellular content [1,8,9].

In this case report, we present a prenatal diagnosis of hemimegalencephaly. Initially, a prenatal ultrasound was performed, the findings of which were mistakenly interpreted as intraparenchymal hemorrhage with unilateral ventriculomegaly. However, a fetal MRI was performed, which allowed for an adequate characterization of the abnormalities and a correct diagnosis.

## Conclusions

Hemimegalencephaly is a rare diagnosis that in most cases is made postnatally. This case illustrates the prenatal imaging findings on ultrasound and MRI, highlighting the difficulties in correctly interpreting the ultrasound findings in those who are not familiar with this condition and emphasizing the importance of fetal MRI as a confirmatory test. In addition, it underscores the importance of early diagnosis to provide counseling to parents and plan for treatment.
